# Targeting fibroblasts in pathological bone formation: mechanisms and treatments

**DOI:** 10.3389/fcell.2025.1612950

**Published:** 2025-05-26

**Authors:** Qianyu Zhang, Qimin Song, Zeyin Li, Xinyi Wu, Yuxiong Chen, Hui Lin

**Affiliations:** ^1^ School of Basic Medical Sciences, Jiangxi Medical College, Nanchang University, Nanchang, China; ^2^ Queen Mary School, Nanchang University, Nanchang, China

**Keywords:** fibroblasts, pathological bone formation, heterotopic ossification (HO), ankylosing spondylitis (AS), ossification of the posterior longitudinal ligament (OPLL)

## Abstract

Fibroblasts are integral to the pathological processes underlying abnormal bone formation, including heterotopic ossification (HO), ankylosing spondylitis (AS), and ossification of the posterior longitudinal ligament (OPLL). This review summarized the diverse roles of fibroblasts, from their transdifferentiation into osteoblast-like cells to their influence on inflammatory and mechanical signal transduction pathways, including those mediated by BMP, TGF-β, and Wnt/β-catenin. In particular, senescent fibroblasts can secrete Activin A to activate the BMP pathway to drive HO formation, and fibroblasts can also differentiate into osteoblasts via interactions among the TGF-β1, BMP-2, and FGF-2 pathways. In AS and OPLL, fibroblasts respond to inflammatory signals and mechanical stress, contributing to pathological bone formation through extracellular matrix remodeling and osteogenic gene expression. In rare cases, fibroblast-mediated abnormal ossification also occurs in diffuse idiopathic skeletal hyperostosis (DISH) and systemic sclerosis (SSc). Therapeutic strategies targeting fibroblast signaling pathways, inflammation, and senescence are highlighted as potential interventions to mitigate these conditions.

## 1 Introduction

Fibroblasts, traditionally viewed as spindle-shaped cells embedded in connective tissues, play pivotal roles in tissue homeostasis, repair, and fibrosis, and were once considered a uniform population (Schuster et al., 2021; Boraldi et al., 2024). Two universal fibroblast transcriptional subtypes (Pi16+ and Col15a1+) exhibit cross-species conservation, being present in both mouse and human tissues, and may function as a reservoir that gives rise to specialized fibroblasts (steady-state) in homeostatic tissues and activated fibroblasts (perturbed-state) under certain pathological conditions (injury, infection, and arthritis) which play a crucial role in extracellular matrix (ECM) (Buechler et al., 2021). These activated fibroblasts along with other cells including chondrocytes and osteoblasts, can synthesize and secrete ECM, contributing to structural support and facilitating tissue repair (Schuster et al., 2021; Buechler et al., 2021). Activated fibroblasts can aberrantly drive excessive ECM deposition and often mineralization, thereby promoting pathological bone formation. Among these conditions, HO is the most prevalent disorder, characterized by the ectopic formation of bone tissue within soft tissues (Jin et al., 2023). Additionally, activated fibroblasts are also associated with other forms of pathologic bone formation including AS and OPLL (Yan et al., 2024; Shi et al., 2019). In rare cases, fibroblasts can also be induced to undergo abnormal ossification in DISH and SSc (Kuperus et al., 2020; Davuluri et al., 2022).

HO is defined as the formation of mature lamellar bone within soft tissues outside the normal skeleton (Xie et al., 2024; Huang et al., 2021). HO can be classified into Acquired HO and Genetic HO (Hwang et al., 2022). Acquired HO, or traumatic HO (tHO), is the most common form (Cao et al., 2023), with an incidence exceeding 50% in femoral fracture patients and 65% in those with explosive injuries. While its exact mechanisms remain unclear (Davuluri et al., 2022), it is believed to result from fibroblast and macrophage recruitment to inflammatory sites, contributing to osteogenesis (Li et al., 2023). Abnormal transdifferentiation of progenitor cells into osteoblasts has also been observed in injuries (Jin et al., 2023). Genetic HO, though rare, is typically more severe. Fibrodysplasia ossificans progressiva (FOP), a disabling genetic HO, causes progressive immobility and heterotopic endochondral ossifications (Pignolo et al., 2023). Prognosis is poor, with patients often wheelchair-bound by their twenties or thirties and eventually experiencing cardiorespiratory failure due to chest wall fixation (Hwang et al., 2022).

By contrast, AS and OPLL involve aberrant bone formation at specific sites within the axial skeleton. AS is a chronic inflammatory spondyloarthropathy marked by entheseal inflammation and new bone bridging joints that lead to bony ankylosis (Liu et al., 2023). OPLL is characterized by progressive ectopic ossification of the spinal ligaments, and no definitive evidence currently connects it to genetic polymorphisms or particular cell types (Le et al., 2022; Saetia et al., 2011; Yang et al., 2020). Although AS and OPLL produce ectopic bone, these lesions occur in ligamentous or entheseal locations rather than in extraskeletal soft tissues, distinguishing them from classic HO.

In HO itself, fibroblasts have emerged as active drivers of ectopic bone formation in both acquired and genetic HO (Cadinu et al., 2024; Zhang C. et al., 2024). In genetic HO, such as FOP, senescent fibroblasts secrete senescence-associated secretory phenotypes (SASPs) (Wang et al., 2022), including Activin A, which may activate the osteogenic BMP signaling pathway, leading to HO in FOP patients (Zhang et al., 2022). In acquired HO, fibroblasts act as key regulators (Griffin et al., 2020). Upon injury, TGF-β1 activates fibroblasts, turning them into a perturbed state and increasing their sensitivity to FGF-2 (Wang et al., 2023; Song et al., 2002). BMP-2 induces Runx2 expression via Smad 1/5/8, whereas FGF-2 activates the Ras/RAF/MEK/ERK pathway, leading to Runx2 phosphorylation (Jiang et al., 2021; Rahman et al., 2015). This enhances the expression of osteogenic genes such as Collagen type I (COL I), Osteocalcin (OCN), and Alkaline phosphatase (ALP), driving fibroblast differentiation into osteoblasts and promoting HO (Song et al., 2017). Recent advancements in fibroblast heterogeneity have been observed across different species, organs, and developmental stages (Lendahl et al., 2022). Beyond skeletal pathologies, similar principles apply in specific organs. Empirical evidence reveals that cardiac fibroblasts (CFs) exhibit phenotypic plasticity towards osteogenic lineage commitment, mechanistically participating in the pathogenesis of HO in the heart (Pillai et al., 2017).

Similarly, fibroblasts contribute to pathological ossification in AS and OPLL. In AS, resident synovial and AS fibroblasts (ASFs) organize local inflammation and tissue remodeling, and can directly promote new bone formation (Liu et al., 2023). Chronic inflammation reprograms fibroblasts to promotes transdifferentiation via the Wnt/β-catenin, ALP, and BMP2 signaling pathways (Jin et al., 2023). Additionally, increased expression of miRNAs enhances osteogenic activity, promoting mineralization and abnormal bone formation (Qin et al., 2019). Inflammation-induced fibroblast changes, such as Tenascin-C secretion, also contribute to extracellular matrix remodeling and new bone formation (Li et al., 2021). In OPLL, mechanical stress-induced downregulation of vimentin in fibroblasts triggers ossification in the posterior longitudinal ligament (Zhang et al., 2014), whereas Connexin43 (Cx43), a gap junction protein, enhances fibroblast responses to mechanical strain, upregulates osteogenic gene expression and promotes ligament calcification (Yang et al., 2011).

In DISH, a systemic condition characterized by abnormal calcification and ossification of ligaments and entheses (Kuperus et al., 2020), fibroblasts actively contribute to abnormal ossification through growth factors like BMP-2 and TGF-β, leading to pathological bone formation similar to HO. Inflammation and mechanical stress further exacerbate this process (Licini et al., 2020; Mader et al., 2017). In SSc, a chronic autoimmune disease marked by excessive fibrosis affecting the skin and internal organs (Davuluri et al., 2022), fibroblasts primarily drive fibrosis by producing excess collagen and extracellular matrix. Although frank bone formation is uncommon in SSc, chronic inflammation and altered mineral metabolism can result in secondary calcinosis in some patients, indicating that fibroblasts can, in rare cases, contribute to ossification (Botzoris et al., 2009).

Taken together, emerging evidence implicates fibroblasts as central mediators of inflammation-driven and mechanoresponsive bone formation in diverse contexts. In this review, we synthesise current insights into fibroblast-driven mechanisms of aberrant ossification and discuss how targeting fibroblast activation and differentiation might offer new therapeutic opportunities.

## 2 The formation and regulatory mechanisms of HO driven by injury-activated and senescent fibroblasts

### 2.1 Fibroblasts become responsive to cytokines to induce HO after injury

**FIGURE 1:**
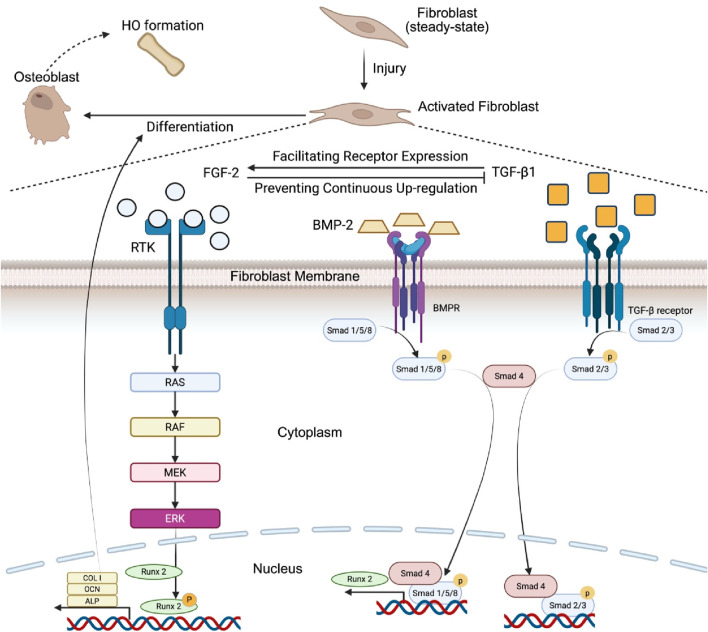
Synergistic interaction between BMP-2, FGF-2, and TGF-β1 in the promotion of heterotopic bone formation. Upon injury, fibroblasts are activated by TGF-β1, increasing their responsiveness to FGF-2. BMP-2 primarily promotes Runx2 expression via Smad 1/5/8 activation. Moreover, FGF-2 stimulates the Ras/RAF/MEK/ERK signaling cascade, leading to the phosphorylation of Runx2 and enhancing its activity. Phosphorylated Runx2 then regulates the expression of osteogenic genes, including COL I, OCN, and ALP, which leads to the differentiation of fibroblasts into osteoblasts, promoting osteogenesis and HO formation.

Injury is one of the preconditions of acquired HO (Shehab et al., 2002). Following an injury, the body triggers an acute inflammatory response through the release of inflammatory-promoting cytokines, which are essential in mediating the inflammation process and the subsequent phases of wound healing ​ (Wautier and Wautier, 2023). Fibroblasts have been observed to respond to these various cytokines which could in terms lead to acquired HO (Yan et al., 2024). Research has documented an immediate increase in TGF-β1 expression in fibroblasts after injury. It is suggested that TGF-β1 may first prime the fibroblasts into perturbed state and increasing their sensitivity to FGF-2 by facilitating receptors expression (Wang et al., 2023; Song et al., 2002), while the later inhibitory effects of elevation of FGF-2 expression in the wound closure process might hinder the sustained upregulation of TGF-β1 in the later stages of wound repair. FGF-2 has been shown to interact with the signaling pathway of BMP-2 to induce the HO (Park et al., 2023). The BMP-2 pathway is crucial for controlling the differentiation of fibroblasts into osteoblasts and the subsequent process of bone formation (Ding et al., 2020). When the downstream molecules of BMP-2 pathway, namely, R-Smads (Smads 1/5/8), become activated and combined with Smad 4, they migrate into the nucleus, the location in which they engage with multiple transcription factors to enhance the expression of Runx2 (Jiang et al., 2021; Rahman et al., 2015). FGF-2 pathway, on the other hand, can phosphorylate Runx2 by activating the Ras/MAPK/ERK pathway (Zhang et al., 2023; Qian et al., 2014). Phosphorylated Runx2 can then alter the expression for osteogenic genes such as ALP, OCN and COL I to promote osteogenesis (Qian et al., 2014; de Farias et al., 2023). Taken together, under injury, fibroblasts become more sensitive to FGF-2 as an effect of increased TGF-β1, FGF-2 then acts synergistically with BMP-2 to promote the development and differentiation of osteoblasts (Ding et al., 2020). BMP-2 pathway first increases the expression for Runx2 (Hwang et al., 2023). FGF-2 then activates Ras/MAPK/ERK pathway to phosphorylate and activate Runx2 to alter the expression of target genes to promote differentiation of osteoblasts and osteogenesis, resulting in HO formation (Zhang et al., 2023; Qian et al., 2014) (Figure 1).

### 2.2 Fibroblasts become senescent cells that induce HO subsequent to external trauma

**FIGURE 2:**
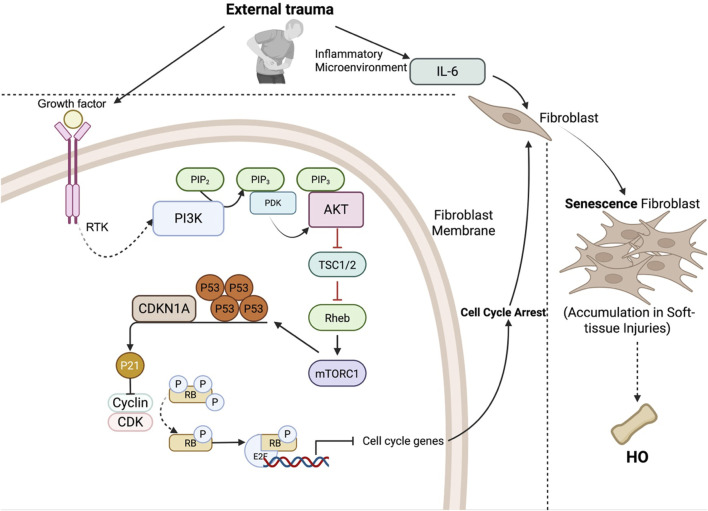
Fibroblast senescence pathway following external trauma. External trauma causes damage to fibroblasts, leading to stress-induced premature senescence (SIPS), which is a classic characteristic of HO. This process, which is mediated by the PI3K/AKT pathway, involves AKT-mediated inhibition of the TSC1/2 complex, activation of mTORC1, and increased p53 synthesis. p53 increases CDKN1A (p21) expression, which inhibits cyclin-CDK complexes, resulting in RB hypophosphorylation. RB binds with E2F and suppresses cell cycle genes, leading to cell cycle arrest. Additionally, elevated IL-6 in the inflammatory microenvironment promotes senescence, leading to the accumulation of senescent fibroblasts in soft tissues and subsequent HO formation.

External trauma, including blunt trauma, illness caused by viral infection, muscle overuse, or toxic chemical exposure, can damage fibroblasts, affecting DNA, cellular organelles, and other structures (Wang et al., 2022). Such trauma may induce dystrophic calcification (DC), characterized by amorphous calcium deposits in fibrocartilaginous tissues, or HO, identified by the presence of woven and lamellar bone structures in soft tissues (Fournier et al., 2020). In the context of HO, fibroblasts enter a state of stress-induced premature senescence (SIPS), becoming injury-induced fibroblasts that promote heterotopic bone formation (Jia et al., 2023), and a greater number of senescent fibroblasts exhibit an activated phenotype marked by cytokine secretion (Mahmoudi et al., 2019). Unlike DCs, which passively accumulate minerals within cells in a degenerative setting, senescent fibroblasts contribute to HO by releasing Activin A, which triggers osteogenic signaling pathways such as BMP to drive the differentiation of nearby cells into osteoblasts. This senescence involves permanent cell-cycle arrest, morphological changes, and the secretion of proinflammatory factors (Zhang et al., 2022; Billimoria and Bhatt, 2023). The process of turning fibroblasts into senescent fibroblasts following injury is mediated by the PI3K/Akt pathway, where the TSC1/TSC2 complex signals through mTORC1 to increase the synthesis of the p53 protein (Astle et al., 2012; Kang et al., 2024). The p53 protein can then bind to the promoter region of CDKN1A to increase its transcription of p21 (Engeland, 2022; el-Deiry et al., 1994). p21 is a cyclin-dependent kinase inhibitor that can subsequently bind to and block the activity of multiple cyclin-CDK complexes, which leads to the hypophosphorylation and inactivation of RB (Engeland, 2022). RB then forms an association with E2F, and the complex binds to the E2 binding sites of the promoters of downstream target genes, leading to their downregulation (Ling and Yang, 2024). Since most of the target genes of the RB-E2F complex are responsible for the progression of the cell cycle, their downregulation leads to cell cycle arrest, which is a classic characteristic of senescent cells (Engeland, 2022; Calcinotto et al., 2019; Sanidas et al., 2024). In addition, the microenvironment is also important in turning fibroblasts into a senescent state. IL-6 is elevated during inflammation and is crucial for the induction of senescence. Trajectory analysis demonstrated that fibroblasts induced by injury differ from those subjected to normal aging. Cells within the same branch shared a similar differentiation state, whereas those in separate branches presented distinct differentiation characteristics. Zhang et al.’s analysis of senescent fibroblasts identified four branches. On day 7, most senescent fibroblasts were found in the root and branch I, whereas by day 21, they were distributed across branches I and II. In contrast, fibroblasts from day 0 and day 42 were predominantly located in branch III, indicating variations in senescent cell types over time. The separation of injury-induced senescence (days 7 and 21) from age-related senescence (days 0 and 42) into different branches suggests divergent differentiation trajectories (Engeland, 2022). Previous studies have shown that these specific senescent cells can accumulate at sites of soft-tissue injury and induce various pathological changes, including HO (Pignolo et al., 2019) (Figure 2).

**FIGURE 3:**
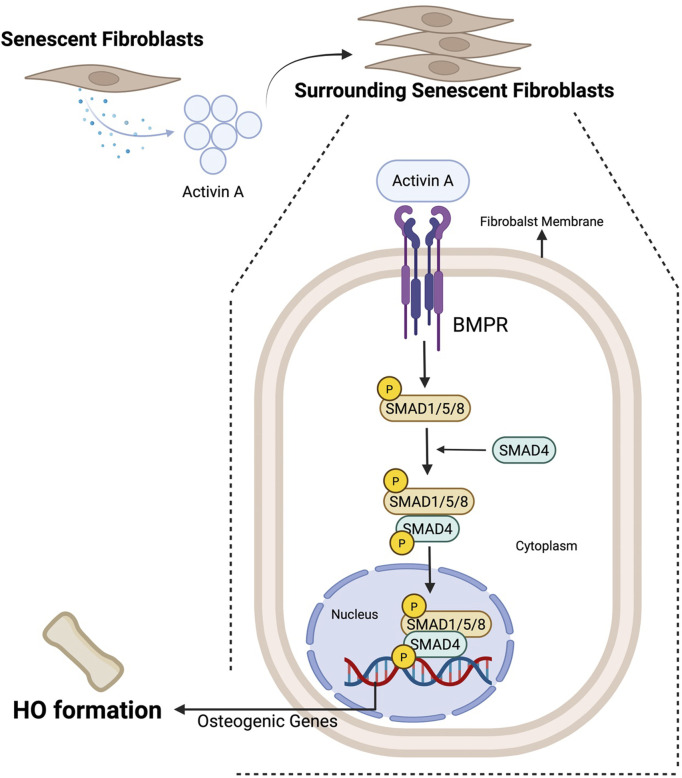
SASP-mediated mechanisms of HO formation by senescent fibroblasts. Senescent fibroblasts secrete SASPs, one of which is activin A, which can activate the BMP pathway, specifically by inducing SMAD1/5/8 phosphorylation, facilitating their interaction with SMAD4 to promote osteogenic gene expression and subsequent HO formation.

Injury-induced senescent fibroblasts cannot directly differentiate into osteogenic cells since they lack the ability to self-replicate and differentiate (Fan et al., 2023). Instead, injury-induced fibroblasts can secrete SASPs, which include numerous cytokines and chemokines (Engeland, 2022; Zhang J. et al., 2024). It is thought to be present as a marker for senescent cells to be recognized by the immune system and thereby eliminated. However, SASPs can also act on the surrounding cells of senescent fibroblasts, potentially leading to the formation of HO through multiple mechanisms. First, SASPs can act on pathways that regulate osteogenesis. For example, in FOP, while it is linked primarily to ACVR1/ALK2 mutations, senescent fibroblasts, which contain a greater proportion of activated fibroblasts—defined by their cytokine secretion—have also been implicated as potential contributors (Mahmoudi et al., 2019). The levels of Activin A, a member of the TGF-β family, are increased (Zhang et al., 2022). Under physiological conditions, Activin A signals through Smad2/3 and is not osteogenic (Srinivasan et al., 2024). However, in FOP patients, Activin A signals through the canonical BMP pathway, leading to the phosphorylation of Smad1/5/8 and increasing the expression of osteogenic genes, thus leading to the development of HO (Hino et al., 2015; Wang et al., 2024) (Figure 3).

## 3 Fibroblast-targeted therapeutic strategies for preventing heterotopic ossification via pathway-specific mechanism inhibition

### 3.1 Inhibiting Senescence-Driven HO via fibroblast mTORC1 blockade

**FIGURE 4:**
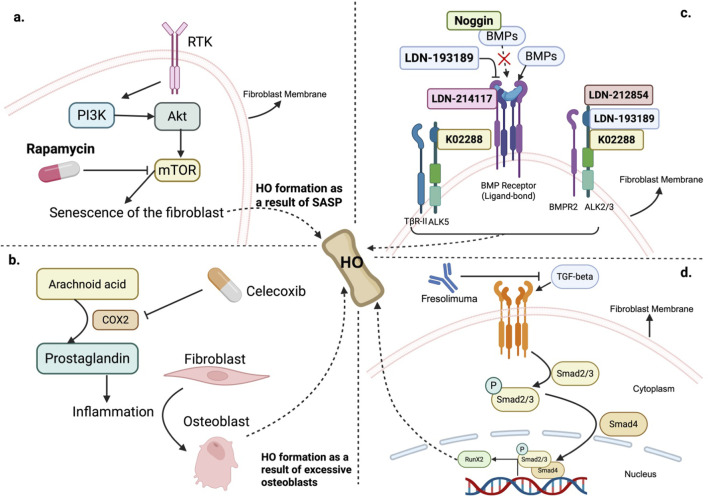
Fibroblast-targeting strategies in HO therapies. **(a)** mTOR activation leads to fibroblast senescence and subsequent HO through the SASP. Rapamycin inhibits mTOR, reducing inflammation and preventing HO development. **(b)** COX-2 inhibition via Celecoxib: Arachidonic acid is metabolized into prostaglandins by COX-2, promoting inflammation and the differentiation of fibroblasts into osteoblasts. Celecoxib, a selective COX-2 inhibitor, suppresses inflammation and reduces HO formation by limiting prostaglandin production and osteoblast activity. **(c)** BMP pathway blockade: BMP signaling, which is mediated by the binding of BMP ligands to BMP receptors (such as BMPR2, ALK2/3, and ALK5), promotes osteogenic differentiation. Therapeutic inhibitors such as LDN-193189, LDN-214117, K02288, and LDN-212854 disrupt this pathway, preventing HO. Noggin inhibits BMP signaling by sequestering BMP ligands, blocking receptor binding, although it can paradoxically activate osteogenesis in the absence of BMP ligands. **(d)** TGF-β pathway blockade: Fresolimumab can bind to all three isoforms of TGF-β to block the downstream TGF-β signaling pathways in fibroblasts, serving as a potential candidate to prevent HO formation.

The mTOR signaling pathway is frequently upregulated by injury-associated inflammation and is a key driver of fibroblast senescence that culminates in HO (Laplante and Sabatini, 2012). In particular, mTORC1 activation converts fibroblasts into senescent cells, thereby promoting HO formation (Astle et al., 2012; Engeland, 2022; el-Deiry et al., 1994; Calcinotto et al., 2019). Mechanism-based intervention using the mTORC1 inhibitor Rapamycin has been shown to downregulate inflammatory mediators such as IL-6, attenuate mTOR signaling, and prevent senescence-associated HO *in vitro* and *in vivo* (Lee et al., 2024; Gundermann et al., 2014). Thus, Rapamycin represents a targeted strategy to halt the senescence pathway in fibroblasts and block subsequent ectopic bone formation. Inhibiting Senescence-Driven HO via Fibroblast mTORC1 Blockade (Figure 4a).

### 3.2 Attenuating inflammation to prevent fibroblast-mediated HO

**FIGURE 5:**
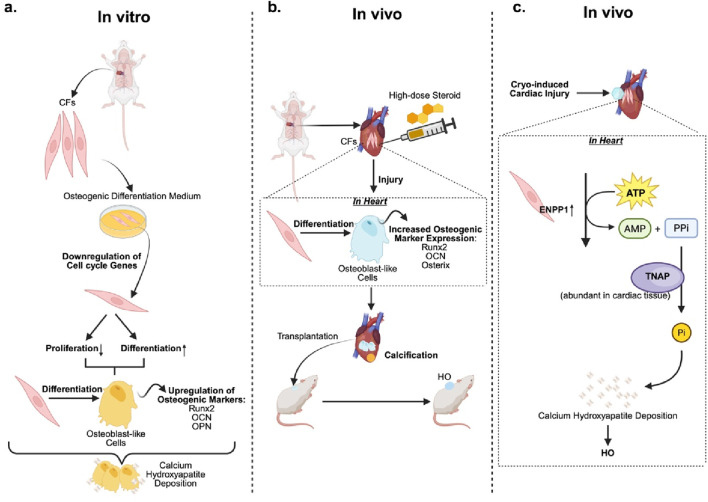
CFs contribute to heart HO through osteogenic differentiation. **(a)** *In vitro*, CFs cultured in osteogenic differentiation medium downregulated the expression of cell cycle genes and upregulated the expression of osteogenic markers, including Runx2, OCN, and OPN, leading to osteoblast-like differentiation and calcium hydroxyapatite deposition. **(b)** *In vivo*, high-dose steroids cause injury and induce CF differentiation in the heart, increasing the expression of osteogenic markers, including Runx2, OCN and Osterix, and contributing to calcification. Transplantation of CFs from the calcified region of the heart to another host’s subcutaneous pocket causes ectopic ossification in another host. **(c)** Cryoinduced cardiac injury increases ENPP1 expression in CFs. ENPP1 breaks down ATP into AMP and pyrophosphate (PPi). PPi is then hydrolyzed by TNAP, which is abundant in the heart, into inorganic phosphate (Pi), which promotes the formation of calcium hydroxyapatite and HO.

Since the activation of the mTORC1 pathway, along with other pathways, such as BMP-2, and the FGF-2 pathways that induce HO are all induced by inflammation following injury in fibroblasts to cause HO formation, attenuating inflammation in the first place could be an effective way to prevent HO induced by fibroblasts. Nonsteroidal anti-inflammatory drugs (NSAIDs) effectively reduce inflammation to suppress the development of HO (Pacifici, 2018; Migliorini et al., 2021). For example, Celecoxib, which is classified as an NSAID and functions as an inhibitor of cyclooxygenase 2 (Cox-2), has demonstrated effectiveness in reducing the formation of HO following surgical trauma in mice through inhibiting the transformation of arachnoid acid into prostaglandin, thereby attenuating inflammation (Astle et al., 2012; Cohen and Preuss, 2024). A clinical trial conducted to evaluate the efficacy of Celecoxib has also yielded some positive results (Lavernia et al., 2014) (Figure 5b). Other NSAIDs, including ibuprofen and indomethacin, have been shown to be similarly effective in preventing HO after Total Hip Arthroplasty (THA) in patients who suffer from joint osteoarthritis as a result of their anti-inflammatory effects. Compared with no prophylaxis, both drugs reduce the risk of HO development in patients who undergo THA (Schneider et al., 2023) (Figure 4b).

### 3.3 Blocking BMP-Mediated osteogenic signaling in fibroblast-driven HO

Fibroblasts, under the stimulation of an inflammatory environment, can activate downstream pathways such as the BMP pathway to promote HO, as mentioned previously (Ding et al., 2020). Studies have shown that inhibiting the BMP pathway effectively prevents the development of HO (Hong and Yu, 2009). Inhibitors targeting BMP receptors or BMP ligands can effectively reduce HO (He et al., 2024). For example, LDN-193189 functions as a broad inhibitor of BMP receptors and has demonstrated its efficacy in animal studies of HO, addressing issues such as vascular calcification, where fibroblast activation facilitates the development of HO (Choi et al., 2024; Nemec et al., 2024). Another compound, K02288, has proven highly selective for BMP receptors over a wide array of human kinases (Yokogami et al., 2024). In addition, Noggin can also bind to BMPs, preventing their interaction with BMP receptors on fibroblasts and osteoblasts, thus inhibiting the osteogenic differentiation and activity that contribute to HO. Senescent fibroblasts can secrete Activin A to activate the mutant ALK2 receptor, contributing to the development of FOP (Zhang et al., 2022). Studies have shown that LDN193189 and LDN-212854 are highly selective for ALK2 over ALK3 and ALK5 and have proven effective in mouse models of HO (Tsugawa et al., 2014; Strong et al., 2021). LDN-214117 displays superior cellular activity and enhanced selectivity for ALK2 over ALK5 (Wu et al., 2023) (Figure 4c).

### 3.4 Targeting fibroblast TGF-β/Smad2/3 signaling to limit osteoprogenitor expansion

The TGF-β/Smad2/3 signaling pathway plays a critical role in the pathogenesis of HO, particularly in the early stages, by promoting the proliferation of osteoprogenitor cells, which are often fibroblastic in nature. Unlike its direct role in fibrosis, TGF-β in HO expands the pool of these progenitor cells at injury sites but inhibits their terminal differentiation into osteoblasts, thereby requiring secondary signals—such as BMPs—to drive osteogenic commitment (Wu et al., 2024). This dual action positions TGF-β as a key initiator of the cellular and inflammatory environment that primes soft tissues for ectopic bone formation, making it a compelling therapeutic target to prevent HO progression (Wang et al., 2018). Clinical studies targeting TGF-β signaling pathways in fibroblasts have been conducted, one of which involves the use of a high-affinity neutralizing antibody called Fresolimumab to target all three isoforms of TGF-β (TGF-β 1, 2, and 3) in fibroblasts. Findings from these studies have shown that Fresolimumab treatment leads to a swift reduction in genes regulated by TGF-β, including biomarkers such as THBS1 and COMP. Moreover, Fresolimumab also suppressed the expression of genes associated with extracellular matrix production, such as connective tissue growth factor (CTGF), validating its role in inhibiting the TGF-β pathway in fibroblasts. Fresolimumab therefore serves as a potential candidate drug to help inhibit the formation of HO by blocking the TGF-β pathway in fibroblasts (Rice et al., 2015) (Figure 4d).

## 4 Classification of fibroblast subpopulations and the molecular mechanisms of CF-Induced HO

Recent advances have highlighted the remarkable heterogeneity of fibroblasts across species, organs, and developmental stages, with multiple distinct subpopulations identified even within the same tissue. In human skin, single-cell RNA sequencing (scRNA-seq) studies have revealed six fibroblast subtypes with distinct gene expression signatures, among which Dipeptidyl Peptidase-4^+^ (DPP4^+^) fibroblasts are the primary producers of ECM components (Vorstandlechner et al., 2020). A comprehensive meta-analysis further classified human skin fibroblasts into ten subtypes, which were consolidated into three major groups: Group A (MMP2^+^), primarily involved in ECM production; Group B (IGFBP7^+^), mainly associated with immune surveillance. In the human lung, five fibroblast subtypes have been characterized, including a Collagen Triple Helix Repeat Containing 1^+^ (CTHRC1^+^) population that is notably enriched in fibrotic regions of COVID-19 patient lungs. Similarly, in the human heart, several CF subtypes have been identified. Among them, two exhibit chamber-specific enrichment—SCN7A^+^ in the atria and CFH^+^ in the ventricles—while subtypes FB4 and FB5 display specialized properties, including heightened responsiveness to TGF-β signaling and active participation in ECM remodeling (Lendahl et al., 2022). Recent studies have further explored the connection between CFs and HO, demonstrating that fibroblasts can contribute to pathological calcification in the heart through changes in cell fate and activation of the ENPP1–PPi–Pi axis.

In mammals, external injuries to the heart can trigger HO, where CFs adopt an osteogenic fate, leading to matrix mineralization and HO formation within the myocardium (Pillai et al., 2017). It is the most common cause of heart block when calcification and fibrosis of the myocardium impedes the propagation of electrical impulses (Lev, 1964). Cardiac calcification is prognostically poor when severe consequences such as myocardial infarction and myocarditis occur (Pillai et al., 2017).

Recently, both *in vitro* and *in vivo* experiments have shown that CFs might contribute to the formation of HO in the heart. In an *in vitro* experiment, CFs were isolated from mice, and after being cultured for a period of time in osteogenic differentiation medium (DM), these CFs produced a deposition of calcium hydroxyapatite, suggesting the plasticity of the CFs (Pillai et al., 2017; Ubil et al., 2014). This kind of osteogenic fate of CFs is characterized by the expression of genes that regulate the cell cycle, which are normally highly expressed and are downregulated to reduce the rate of proliferation. This finding is consistent with the principle that a reduction in proliferation is linked to the induction of differentiation (Buttitta and Edgar, 2007). In contrast, canonical osteoblast marker genes such as Runx2 and OCN as well as extracellular matrix proteins such as OPN are upregulated upon differentiation (Figure 5a). CFs have also been shown to directly participate in HO after injury to the heart, as validated by *in vivo* experiments. Injury induced by a high dose of steroids triggers osteogenic differentiation in CFs. While uninjured hearts presented minimal expression of osteogenic markers (Runx2, OCN, and Osterix), injury caused a significant increase in their expression, especially in the presence of calcified myocardium. When harvested CFs obtained from the calcified myocardium of C3H mice were injected into another host’s subcutaneous pocket, the formation of ectopic ossifications was observed, suggesting that CFs are directly involved in driving the calcification of soft tissue (Pillai et al., 2017) (Figure 5b).

Another potential mechanism for cardiac calcification mediated by CFs following injury involves an enzyme ENPP, which is often expressed in osteoblasts and is associated with osteoblast maturation and bone mineralization (Johnson et al., 2003). Following Cryo-induced cardiac injury, the expression of ENPP1 in CFs increases, and ATP is subsequently broken down by ENPP1, during which pyrophosphate (PPi) is generated on the cell surface together with AMP. PPi is further hydrolyzed into Pi by tissue nonspecific alkaline phosphatase (TNAP), which is abundant in cardiac tissue (Pillai et al., 2017; Rutsch et al., 2011). Pi can continue to support the formation of calcium hydroxyapatite to promote mineralization, eventually leading to HO (Pillai et al., 2017; Johnson et al., 2003) (Figure 5c).

## 5 Pathogenic mechanisms of fibroblast-driven abnormal ossification in AS

AS is an inflammatory disease with a chronic course that affects mainly the pelvis and spine. AS patients often exhibit abnormal bone formation (Jin et al., 2023), which is caused primarily by excessive generation of osteoblasts (Bleil et al., 2016). Fibroblasts are reprogrammed and transdifferentiated into osteoblasts during inflammation, leading to the occurrence of abnormal ossification (Zhang et al., 2020).

### 5.1 ASF-mediated bone erosion and pathological ossification

In AS, activated synovial fibroblasts (ASFs) are pivotal in driving pathological bone formation through interconnected cellular processes and signaling pathways. ASFs not only cause joint damage but actively stimulate pathological bone formation by recruiting osteoblasts, differentiating into other cell types, and directly participating in bone formation (Liu et al., 2023). They affect osteoclast activity, promoting early - stage bone erosion via the secretion of RANKL, a key molecule for osteoclast differentiation and activation. Along with inflammatory cytokines and under Th17 cell influence, RANKL production creates a pro - inflammatory environment that enhances osteoclast function and leads to cartilage and bone erosion, ultimately facilitating the transition from bone resorption to abnormal new bone formation (Sieper et al., 2019). ASFs then recruit osteoblasts to areas near remaining cartilage islands, and the overexpression of BMPs within ASFs, which are powerful regulators of osteoblast differentiation and bone matrix production, enhances osteoblast - mediated bone formation, directly linking ASFs to pathological ossification in AS (Bharadwaz and Jayasuriya, 2021). Additionally, ASFs contribute to tissue composition changes that support abnormal bone formation, such as increased fat presence in ASF - rich tissues, with fat accumulation sometimes seen in a spindle - shaped pattern within ASFs (Bleil et al., 2016). Under BMP influence, ASFs may differentiate into adipocytes, indicating their multipotency and ability to shift the local environment towards supporting tissue remodeling and ossification (Chiowchanwisawakit et al., 2011).

### 5.2 Inflammatory signal-driven fibroblast-to-osteoblast transdifferentiation mechanisms

**FIGURE 6:**
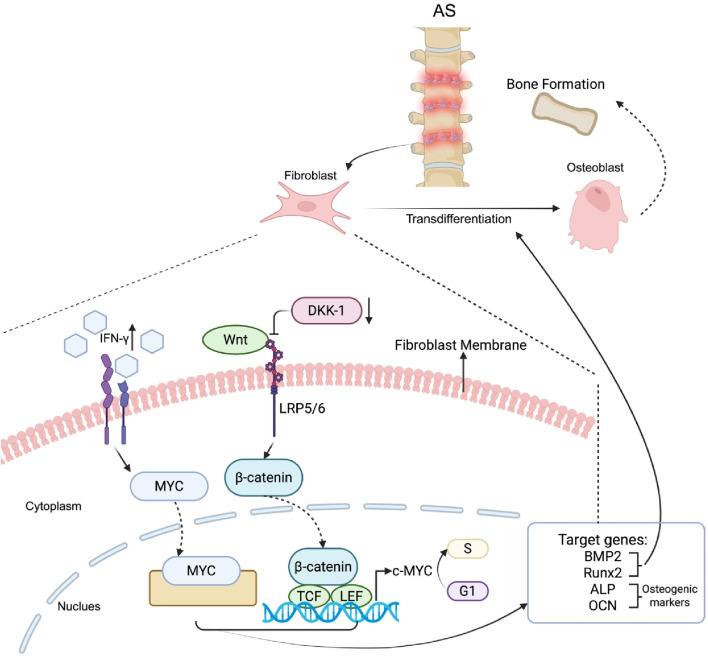
Mechanisms of fibroblast transdifferentiation into osteoblasts in AS. In AS patients, fibroblasts express receptors for inflammatory factors such as IFN-γ, which promotes the upregulation of MYC expression. MYC induces transdifferentiation by increasing the expression of osteogenic markers, including ALP and BMP2. In parallel, inflammation reduces the expression of DKK-1, a Wnt signaling inhibitor, thereby activating the Wnt pathway. β-catenin is then stabilized, migrates to the nucleus and forms a complex with TCF/LEF transcription factors to regulate the expression of osteogenic genes such as Runx2, ALP, OCN, and BMP2. Collectively, these pathways promote the conversion of fibroblasts into osteoblasts.

During inflammation, fibroblasts can express receptors for various inflammatory factors, such as IFN-γ; when fibroblasts are excessively stimulated by IFN-γ, the expression of the MYC gene is elevated. MYC is known to induce transdifferentiation and promote the conversion of fibroblasts to osteoblasts via the upregulation of ALP and BMP2 expression (Jin et al., 2023) (Figure 6). The roles of ALP and BMP2 in transdifferentiating fibroblasts into osteoblasts have also been validated in human gingival fibroblasts (HGFs). By using 5-aza-2′-deoxycytidine (5-aza-dC) to demethylate CpG islands in ALP and Runx2 and subsequently stimulating HGFs with BMP2 to promote the expression of ALP and Runx2 in the hypomethylated stage, HGFs successfully transdifferentiate into the osteoblast lineage (Cho et al., 2017).

Another potential mechanism of transdifferentiating fibroblasts into osteoblasts during inflammation is via the Wnt signaling pathway (Zou et al., 2016). The Wnt signaling pathway is also well recognized in AS patients (Hong and Zhang, 2020). The activity of the Wnt signaling pathway is elevated due to the reduction in DKK-1 (a Wnt inhibitor) in fibroblasts during inflammation (Haynes et al., 2012). The canonical Wnt pathway involves regulating the intracellular expression of β-catenin, which subsequently regulates genes that are responsible for osteoblast formation (Zou et al., 2016; Xu et al., 2022). For example, c-myc is one of the effectors downstream of the Wnt signaling pathway, and its expression is elevated following Wnt signaling pathway activation. Upon elevation, c-myc responds to mitogenic signals to promote the progression of the cell cycle from G1 to S phase, which leads to an increased proliferation rate of fibroblasts (Spencer and Groudine, 1991). In addition, osteogenic genes such as Runx2, ALP and OCN are downstream targets of the Wnt/β-catenin pathway, where Runx2 enhances osteoblast differentiation (Song et al., 2023), and ALP and OCN function as osteogenic markers (Day et al., 2005) (Figure 6).

### 5.3 Fibroblast-associated molecular regulators and targeted therapeutic strategies

Fibroblasts can promote abnormal bone formation in AS due to their enhanced osteogenic differentiation. In AS patients, fibroblasts derived from ligament tissues exhibit high levels of miR-17-5p, which promotes osteogenic activity by increasing the expression of markers, including COL1A1, RUNX2, BMP2, and ALP activity, and increasing mineralization. Mechanistically, miR-17-5p directly interacts with ANKH, a key regulator of pyrophosphate transport that normally inhibits mineralization. The suppression of ANKH caused by miR-17-5p reduces extracellular pyrophosphate levels, thereby accelerating ectopic mineralization and ossification. Additionally, miR-17-5p modulates cytokines by suppressing DKK-1 (an inhibitor of the Wnt pathway) and increasing VEGF, further encouraging osteogenic differentiation through activation of the Wnt signaling pathway. In an AS rat model, inhibition of miR-17-5p effectively reduced osteophyte formation, alleviated sacroiliitis, and slowed disease progression, suggesting that targeting miR-17-5p could serve as a promising therapeutic strategy to prevent fibroblast-driven HO in AS (Qin et al., 2019).

Fibroblasts isolated from AS patients exhibit increased osteogenic activity when exposed to low concentrations of TNF-α, a proinflammatory cytokine. miR-21 expression is significantly elevated under these conditions, leading to increased expression of osteogenic markers such as Runx2, BMP2, OPN, and osteocalcin. Mechanistically, miR-21 is closely linked to the JAK2/STAT3 pathway, where STAT3 activation and nuclear translocation stimulate miR-21 expression, forming a positive feedback loop. Exogenous overexpression of miR-21 in proteoglycan-induced arthritis (PGIA) mouse models induces new bone formation and sacroiliac joint (SIJ) fusion, which is facilitated through the JAK2/STAT3 signaling cascade and elevated IL-17 levels. These findings suggest that miR-21 acts as a critical mediator connecting inflammation to abnormal bone formation in AS, suggesting potential therapeutic targets for managing fibroblast-driven HO (Zou et al., 2020).

In response to inflammation, fibroblast-specific protein-1 (FSP1)+ fibroblasts in entheses secrete excessive Tenascin-C (TNC), which remodels the ECM by suppressing adhesion forces. This reduction in adhesion force activates the Hippo signaling pathway and leads to yes-associated protein (YAP) phosphorylation, preventing its nuclear translocation. Consequently, this activates chondrogenic differentiation, promoting the formation of cartilage templates during endochondral ossification, a key mechanism of new bone formation in AS. Experimental inhibition or genetic ablation of TNC in animal models significantly suppresses new bone formation, highlighting its essential role in fibroblast-driven pathological ossification. Thus, inflammation-induced TNC expression by fibroblasts and its mechanotransductive effects on the ECM are central to the progression of HO in AS (Li et al., 2021).

Tofacitinib, an oral JAK inhibitor, could serve as a possible candidate for the treatment of AS by selectively inhibiting JAK1/2/3 (Nakashima et al., 2022), which are involved in cytokine signaling pathways. This mechanism blocks pathways mediated by cytokines such as IL-17, which are implicated in the pathogenesis of AS. By modulating immune responses and reducing inflammation, tofacitinib effectively targets both systemic and local inflammation (van der Heijde et al., 2017). By modulating immune responses and reducing inflammation, tofacitinib effectively targets both systemic and local inflammation (van der Heijde et al., 2017).

## 6 Fibroblast-mediated mechanotransduction and molecular pathways driving OPLL

### 6.1 Mechanical stress-induced vimentin downregulation and osteogenic marker upregulation in OPLL fibroblasts

OPLL refers to a progressive disorder in which abnormal bone growth occurs mostly in the cervical spinal ligament (Nam et al., 2019). Patients with OPLL develop neurological symptoms, ranging from discomfort to severe myelopathy, due to spinal cord and nerve root compression by calcified PLL (Le et al., 2022; Yan et al., 2017). Fibroblasts are involved in the progression of OPLL through mechanical stress-induced changes in gene expression. Specifically, vimentin, an intermediate filament protein, appears to be a key regulator during this process. In OPLL, fibroblasts subjected to mechanical stress show significant downregulation of vimentin expression. This downregulation is a critical factor in the progression of OPLL in the sense that, after the reduction in vimentin following mechanical stress, there is an increase in the expression of osteogenic markers such as OCN, ALP, and COL I, suggesting that the mechanical stress in OPLL fibroblasts might trigger a shift toward osteogenesis, thereby contributing to the ossification process that characterizes OPLL. Experiments using siRNA interference to target vimentin in OPLL fibroblasts confirmed the subsequent elevation of OCN, ALP and COL I, validating the role of vimentin reduction in OPLL fibroblasts following stimulation with mechanical stress (Zhang et al., 2014).

### 6.2 BMAL1 deficiency, cellular senescence, and TGF-β/BMP pathway activation in ligament fibroblasts

Brain and Muscle Aryl Hydrocarbon Receptor Nuclear Translocator-like protein 1 (BMAL1) acts as a transcription factor that is also associated with fibroblasts in OPLL development. It is often responsible for regulating the circadian system and organisms’ interactions with their surrounding environment. Once BMAL1 is deficient, the circadian behavior of the organism will be abnormally altered along with disruption of its interactions with the surroundings, representing a stage of senescence (Storch et al., 2002). The expression level of BMAL1 is reportedly downregulated in human fibroblasts obtained from tendons and ligaments of OPLL patients (Liang et al., 2022; Pignolo et al., 2024). In addition, validation experiments demonstrated that by knocking out Bmal1 in a mouse model, progressive abnormal ossifications were observed. Moreover, senescence significantly influences Bmal1 expression. Specifically, Bmal1 mRNA levels are markedly lower in PLLs and Achilles tendons acquired from 32-week-old mice than in those acquired from 6-week-old mice. In addition, in fibroblasts obtained from the PLL of Bmal1-deficient mice, genes related to the TGFβ/BMP pathway, such as SMAD1/2/3, are elevated compared with those in wild-type mice (Diederichs et al., 2024). The TGFβ/BMP pathway is considered to be a key factor in the progression of pathological ossification. When TGFβ and BMP bind to their type I and type II receptors, downstream Smads are phosphorylated and combine with Smad4 to be translocated to the nucleus and increase Runx2 expression to regulate osteogenesis (Pang et al., 2022). It was therefore concluded that the downregulation of Bmal1 as a result of senescence could activate the TGFβ/BMP pathway to induce osteogenic differentiation of fibroblasts, thus eventually resulting in OPLL (Liang et al., 2022).

### 6.3 Cx43-dependent gap junction signaling and ERK1/2-p38 MAPK–Mediated osteoblastic differentiation

**FIGURE 7:**
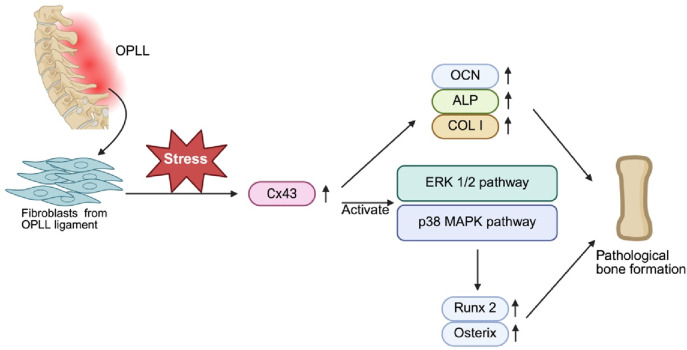
Role of Cx43 in Mechanical Stress-Induced Osteogenesis in OPLL Fibroblasts. Mechanical stress promotes pathological bone formation associated with OPLL by upregulating Cx43 in fibroblasts derived from OPLL spinal ligaments. Increased expression of Cx43 increases the expression of osteogenic markers such as OCN, ALP, and COL I. In addition, ERK1/2 and p38 MAPK are also activated by Cx43, and these pathways also induce the expression of the key transcription factors Runx2 and Osterix, driving the osteoblastic differentiation of ligament fibroblasts and leading to pathological bone formation.

Another potential mechanism by which fibroblasts induce OPLL is via Cx43 (Yang et al., 2011), which is a type of connexin protein that forms gap junctions between cells. As the most prevalent connexin in osteoblasts, it promotes cell-to-cell communication within tissues, including bone. Cx43 is therefore essential in processes such as bone mineralization and the regulation of osteoblast functions, as it helps cells respond to both systemic and local signals, promoting bone formation and repair. These findings suggest that Cx43 might influence the signaling mechanisms triggered by mechanical strain, which is thought to contribute to OPLL development. To test this hypothesis, Yang et al. conducted a study in which fibroblasts from the spinal ligaments of both OPLL patients and healthy individuals were sampled and cultured. Mechanical strain was applied to both groups of fibroblasts, and the expression levels of osteogenic genes, including OCN, ALP and COL I, along with Cx43, were measured at 12 and 24 h after strain application. The results showed that in OPLL cells, mechanical strain led to significant upregulation of the OCN, ALP, COL I, and Cx43 proteins. However, no such changes were observed in fibroblasts from healthy individuals after the strain was applied. When researchers targeted Cx43 with RNA interference in OPLL fibroblasts, they reported that mechanical strain no longer caused any noticeable changes in the expression of OCN, ALP, COL I, or Cx43. These findings suggest that the increase in Cx43 expression induced by mechanical strain in OPLL fibroblasts could facilitate the progression of OPLL through the upregulation of osteogenic genes, which suggests that Cx43 is a key mediator of how OPLL fibroblasts respond to mechanical stress to cause pathological bone formation (Yang et al., 2011). (Figure 7).

Cx43 has also been demonstrated to be essential in the development of OPLL by mediating the mechanical signal transduction that drives the osteoblastic differentiation of ligament fibroblasts. Mechanical stress upregulates Cx43, which in turn activates the ERK1/2 and p38 MAPK signaling pathways, leading to the expression of osteogenic markers such as Runx2 and Osterix, which are key factors in bone formation. Knocking down Cx43 reduces the activation of these pathways and almost completely inhibits the osteogenic effect of mechanical stress (Figure 7). Although the JNK pathway is also activated in response to mechanical stress, it appears to have a minimal impact on osteoblastic differentiation, indicating that ERK1/2 and p38 MAPK serve as the primary drivers of this process (Chen et al., 2016).

### 6.4 Anti-inflammatory therapeutic strategies targeting fibroblast activation in OPLL

Fibroblasts release cytokines, growth factors (such as TGF-β and BMPs), and ECM components, which promote ligament ossification and spinal cord compression. NSAIDs inhibit cyclooxygenase (COX) enzymes, reducing the production of proinflammatory mediators such as prostaglandins. This inhibition can decrease inflammatory signaling and potentially limit fibroblast activation and the subsequent ossification process. Mechanical stress in OPLL can exacerbate inflammation, further activating fibroblasts. By reducing inflammation, NSAIDs might also mitigate the effects of mechanical stress on fibroblast activation, indirectly curbing the progression of ligament ossification. While NSAIDs are primarily used to manage pain symptoms in OPLL, their anti-inflammatory effects could complement other therapeutic strategies aimed at fibroblast modulation, such as targeting the TGF-β or BMP signaling pathways (Tsuchiya and Shinomiya, 2008).

## 7 Fibroblast-mediated mechanisms of potential pathological bone formation

### 7.1 Fibroblast-driven abnormal ossification of ligaments and entheses in DISH

DISH involves fibroblasts in abnormal ossification of ligaments and entheses, driven by genetic factors like ENPP1 mutations that reduce PPi levels, promoting mineralization (Kato et al., 2022). Similar to HO, growth factors (BMP-2, TGF-β) may push fibroblasts to form bone or an osteogenic ECM, while inflammation and mechanical stress activate them, mirroring HO triggers (Licini et al., 2020; Mader et al., 2017). Fibroblasts also alter the ECM, with high decorin staining in ossified tissues, as seen in HO (Licini et al., 2020). Though DISH’s systemic nature and genetic basis differ, its fibroblast-mediated mechanisms—via growth factors, inflammation, and mechanical stress—closely resemble those in HO.

### 7.2 Potential for fibroblast-mediated ossification in systemic sclerosis

SSc is a fibrosing disease driven by overactive dermal and visceral fibroblasts, but frank bone formation is uncommon (Rosendahl et al., 2022; Jinnin, 2010). Instead, SSc fibroblasts produce excessive collagen and ECM, leading to dermal fibrosis and vascular rarefaction. However, secondary calcinosis (calcium deposition) occurs in ∼20–40% of SSc patients. This calcinosis likely reflects a chronically inflamed, hypoxic connective tissue “primed” for mineral deposition. Prolonged inflammation and dysregulated phosphate/pyrophosphate metabolism (involving FGF-23) have been implicated in SSc calcinosis (Davuluri et al., 2022). While SSc fibroblasts do not typically become osteoblasts, their fibrotic matrix provides a substrate for dystrophic calcification. Thus, in SSc, fibroblasts are key effectors of fibrosis, but existing studies also note that in rare cases the fibrotic tissue can ossify (Botzoris et al., 2009).

## 8 Conclusion

Fibroblasts participate in the development of pathological bone formation in various conditions, including HO, AS, OPLL, DISH, and SSc. Their unique plasticity allows them to respond to environmental cues, such as inflammation, injury, and mechanical stress, driving processes that lead to pathological ossification. In HO, injury triggers an inflammatory response, releasing cytokines such as TGF-β1 that sensitize fibroblasts to FGF-2. FGF-2 collaborates with BMP-2 to increase Runx2 expression through the BMP-2 pathway, while the Ras/MAPK/ERK pathway phosphorylates Runx2, activating osteogenic genes (e.g., ALP, OCN, and COL I). This process drives fibroblasts to differentiate into osteoblasts, leading to ectopic bone formation. These coordinated actions highlight the central role of fibroblasts in shaping the pro-ossification niche and driving HO progression.

Fibroblasts are also involved in other types of pathological bone formation. In AS, chronic inflammation drives fibroblast transdifferentiation into osteoblast-like cells through pathways such as the Wnt/β-catenin and JAK2/STAT3 pathways. Key inflammatory mediators such as IFN-γ and miRNAs such as miR-17-5p and miR-21 enhance fibroblast-driven osteogenesis, contributing to ectopic mineralization, joint fusion, and structural rigidity. In OPLL, mechanical stress induces the downregulation of vimentin in fibroblasts, causing the subsequent elevation of osteogenic genes such as OCN, ALP, and COL I, leading to ossification. Additionally, the downregulation of BMAL1 as a result of senescence in OPLL fibroblasts is associated with the activation of the TGF-β/BMP pathway by increasing the expression of downstream molecules. Moreover, Cx43 is linked to mechanical strain, promoting osteoblast differentiation and abnormal ligament ossification. Fibroblasts are also key mediators in driving osteogenic differentiation in DISH (akin to HO), generating a calcification-prone matrix in SSc, and, in rare cases, giving rise to HO-like bone formation.

Given their central role in pathological bone formation, fibroblasts present an attractive therapeutic target. Strategies such as inflammation suppression with NSAIDs or rapamycin, as well as pathway-specific inhibitors like LDN-193189, fresolimumab, and tofacitinib, have shown potential therapeutic effects. These therapies aim to block key signaling pathways or reduce fibroblast activation, thereby mitigating HO-type pathological ossification.

Future research should focus on advancing our understanding of fibroblast-driven processes and refining therapeutic approaches. By targeting the cellular and molecular mechanisms underlying fibroblast activity, it may be possible to develop effective interventions for HO, AS, OPLL, DISH, and pathological ossification in manifestations in SSc, improving patient outcomes and quality of life.
